# Tetrahydroxystilbene Glucoside Regulates Proliferation, Differentiation, and OPG/RANKL/M-CSF Expression in MC3T3-E1 Cells via the PI3K/Akt Pathway

**DOI:** 10.3390/molecules23092306

**Published:** 2018-09-10

**Authors:** Ying-Sai Fan, Qin Li, Nawras Hamdan, Yi-Fei Bian, Shen Zhuang, Kai Fan, Zhong-Jie Liu

**Affiliations:** 1 College of Veterinary Medicine, China Agricultural University, Beijing 100193, China; funinside@cau.edu.cn (Y.-S.F.); nawras_hamdan@hotmail.com (N.H.); bianshuais@163.com (Y.-F.B.); zhuangshenzs@163.com (S.Z.); dirtyfan@sina.com (K.F.); 2 College of Life Science and Food Engineering, Hebei University of Engineering, Handan 056038, China; liqin_00753@outlook.com

**Keywords:** tetrahydroxystilbene glucoside, osteoblast, PI3K/Akt, herbal medicine, osteoporosis

## Abstract

Tetrahydroxystilbene glucoside (TSG) is a unique component of the bone-reinforcing herb *Radix Polygoni Multiflori* Preparata (RPMP). It has the ability to promote bone formation and protect osteoblasts. However, the underlying mechanism remains unclear. To better understand its biological function, we determined TSG’s effect on murine pre-osteoblastic MC3T3-E1 cells by the MTT assay, flow cytometry, FQ-PCR, Western blot, and ELISA. The results showed that TSG caused an elevation of the MC3T3-E1 cell number, the number of cells in the S phase, and the mRNA levels of the runt-related transcription factor-2 (Runx2), osterix (Osx), and collagen type I α1 (Col1a1). In addition, the osteoprotegerin (OPG) mRNA level was up-regulated, while the nuclear factor-κB ligand (RANKL) and macrophage colony-stimulating factor (M-CSF) mRNA levels were down-regulated by TSG. Furthermore, TSG activated the phosphoinosmde-3-kinase/protein kinase B (also known as PI3K/Akt) pathway, and blocking this pathway by the inhibitor LY-294002 could impair TSG’s functions in relation to the MC3T3-E1 cells. In conclusion, TSG could activate the PI3K/Akt pathway and thus promote MC3T3-E1 cell proliferation and differentiation, and influence OPG/RANKL/M-CSF expression. TSG merits further investigation as a potential therapeutic agent for osteoporosis treatment.

## 1. Introduction

Osteoporosis—a devastating systemic skeletal disorder—increases the fracture risk of patients in all populations, resulting in physical, psychosocial, and economic burdens [1]. There are different kinds of modern medicines that alleviate osteoporosis, but they all pose some side effects after long-term administration [2].

Based on the “kidney governing bones” theory in traditional Chinese medicine, the essence stored in kidney promotes and regulates bone growth, and osteoporosis is believed to be mainly caused by kidney deficiency [3]. Many kidney-reinforcing herbs could treat osteoporosis effectively, while posing few side effects [3,4]. Therefore, new effective drugs could be developed from components of these herbs for the treatment of osteoporosis. 

**Figure 1:**
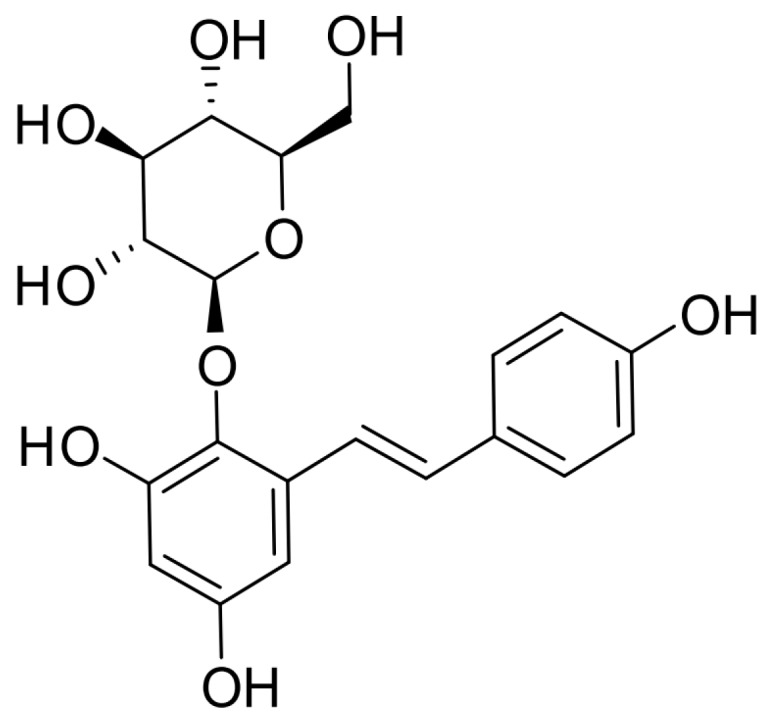
Chemical structural formula of tetrahydroxystilbene glucoside (TSG) [8].

*Radix Polygoni Multiflori* Preparata (RPMP)—the processed root of *Polygonum Multiflorum* Thunb—is valued for its capacity to tonify the kidney and liver and strengthen tendons and bones [5]. Tetrahydroxystilbene glucoside (2,3,5,4′-tetrahydroxystilbene-2-*O*-β-d-glucoside, TSG) (shown in Figure 1) is the unique constituent of RPMP, and has the ability to protect ostoblasts from some pathogenic factors such as dexamethasone, oxidative stress, and inflammation [6,7,8]. However, there has been little research reporting the action of TSG on osteoblast lineage cells under no pathogenic factor affection, and the signaling pathways involved in TSG’s action are still unknown.

Recently, there has been increasing interest in how natural components regulate bone remodeling through osteoblasts. During adulthood, bone is remodeled continuously through osteoblastic bone formation and osteoclastic bone resorption, which bring about dynamic changes in bone size and shape. Osteoporosis occurs when the bone resorption rate is greater than the bone formation rate [2]. In the bone remodeling process, the osteoblast can directly form new bone and indirectly affect the osteoclast absorbing bone by expressing various proteins [9]. For example, for osteoblastic bone formation, runt-related transcription factor-2 (Runx2) and osterix (Osx) are important transcription factors which promote osteoblast differentiation and bone matrix protein production [10], and collagen type I α1 (Col1a1) is a major matrix protein, representing about 90% of the bone organic matrix [9]. For bone resorption, osteoprotegerin (OPG), nuclear factor-κB ligand (RANKL), and macrophage colony-stimulating factor (M-CSF) are proteins that are critical to osteoclast differentiation and viability [11,12]. In addition, there are multiple signaling networks regulating osteoblast growth and function. Among them, the phosphoinosmde-3-kinase/protein kinase B (also known as PI3K/Akt) pathway is a major signaling pathway affecting osteoblast viability and cytokine expression [13,14].

To explore the mechanism of TSG’s action on osteoblasts, we treated MC3T3-E1 cells with TSG, then measured the cell proliferation, cell cycle distribution, and proteins that affected osteoblastic bone formation or osteoclastic bone resorption. Moreover, the role that PI3K/Akt plays in TSG’s action was also tested.

## 2. Results

### 2.1. Effect of TSG on MC3T3-E1 Cell Proliferation and Cell Cycle Distribution

**Figure 2:**
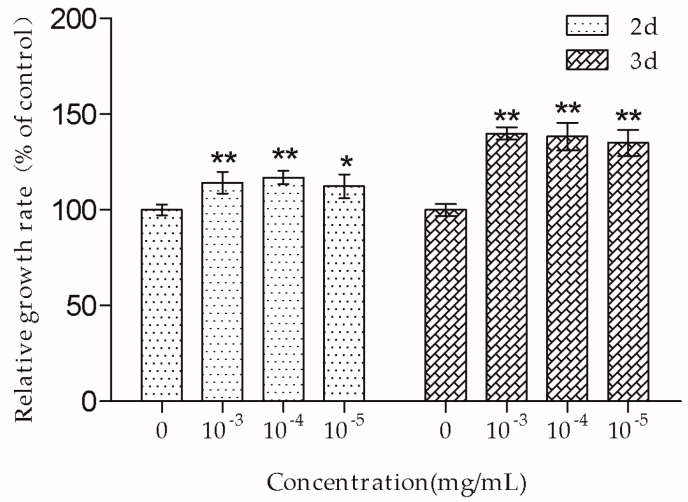
TSG promoted MC3T3-E1 cell proliferation. Data are represented as the mean ± SD of four determinations. * *p* < 0.05 and ** *p* < 0.01 when compared with the control group.

Cell growth rate was examined by the MTT assay. As shown in Figure 2, TSG (10^−3^, 10^−4^, and 10^−5^ mg/mL) significantly increased the number of MC3T3-E1 cells, and the cell growth rate was higher in the groups treated with TSG for three days than those treated for two days.

**Figure 3:**
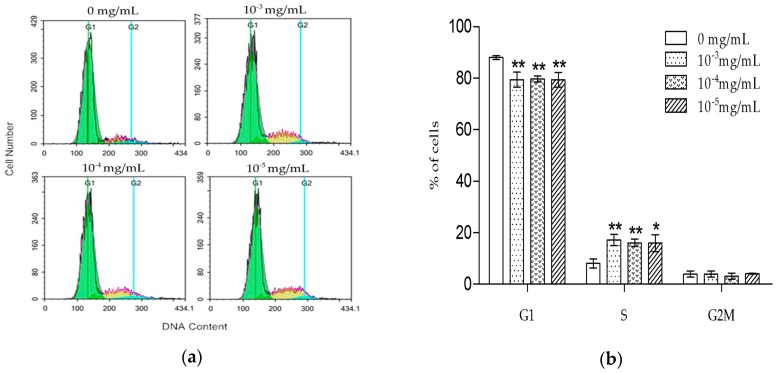
Effect of TSG on (**a**) the DNA content and (**b**) the cell cycle distribution of MC3T3-E1 cells. Data are represented as the mean ± SD of three determinations. * *p* < 0.05 and ** *p* < 0.01 when compared with the control group.

Next, we measured TSG’s effect on MC3T3-E1 cell cycle progression. After being treated with TSG for one day, the cells were stained by PI and tested by flow cytometry. As shown in Figure 3a,b, TSG (10^−3^, 10^−4^, and 10^−5^ mg/mL) increased the percentage of cells in the S phase and decreased the percentage of cells in the G1 phase, indicating that cell DNA synthesis was promoted by TSG.

### 2.2. TSG Promoted MC3T3-E1 Cell Differentiation

Osteoblast differentiation is essential for bone matrix formation. After treating cells with TSG for three days, we measured the mRNA levels of Runx2, Osx, and Col1a1 to determine TSG’s effect on cell differentiation and collagen synthesis.

**Figure 4:**
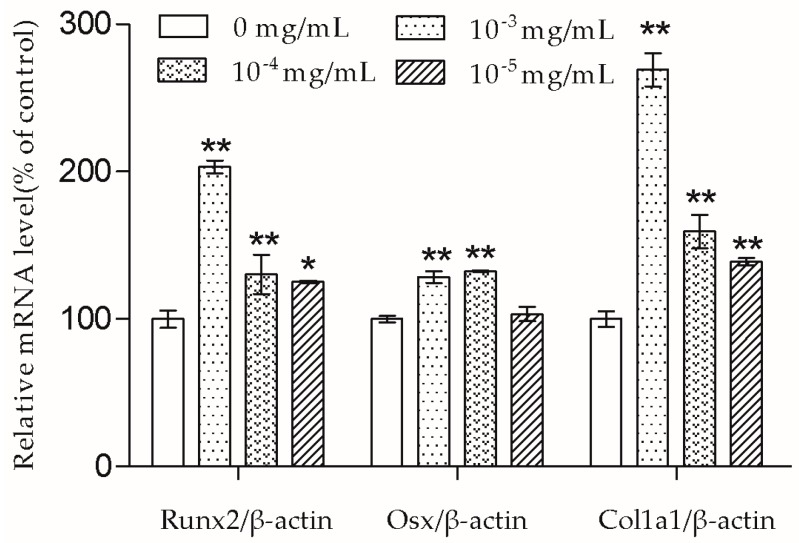
TSG up-regulated runt-related transcription factor-2 (Runx2), osterix (Osx), and collagen type I α1 (Col1a1) mRNA levels of the MC3T3-E1 cells. Data are represented as the mean ± SD of three determinations. * *p* < 0.05 and ** *p* < 0.01 when compared with the control group.

As shown in Figure 4, TSG (10^−3^ and 10^−4^ mg/mL) significantly increased Runx2, Osx, and Col1a1 mRNA levels; TSG (10^−5^ mg/mL) significantly increased Runx2 and Col1a1 mRNA levels, while having no significant effect on the Osx mRNA level.

### 2.3. TSG Regulated OPG, RANKL, and M-CSF mRNA Levels

Osteoblasts regulate osteoclast activity and function by releasing proteins such as OPG, RANKL, and M-CSF, thus influencing bone resorption [11,12]. RANKL and M-CSF promote osteoclast differentiation and activity, while OPG inhibits RANKL’s effect on osteoclasts [11].

**Figure 5:**
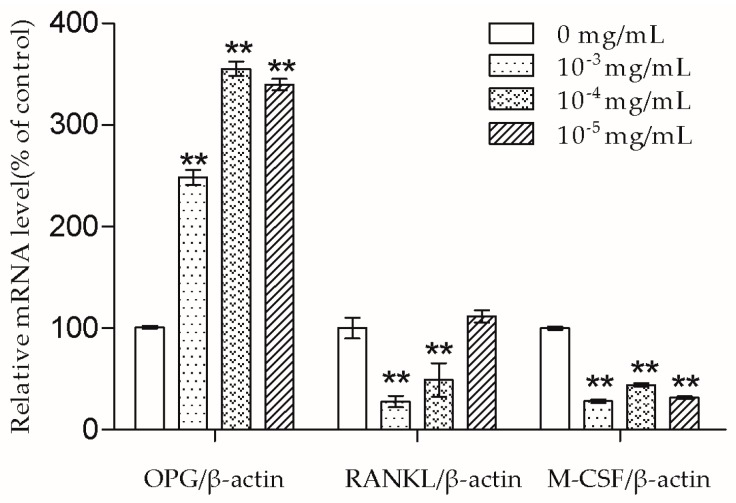
TSG up-regulated the osteoprotegerin (OPG) mRNA level, and down-regulated the nuclear factor-κB ligand (RANKL) and macrophage colony-stimulating factor (M-CSF) mRNA levels of the MC3T3-E1 cells. Data are represented as the mean ± SD of three determinations. ** *p* < 0.01 when compared with the control group.

As shown in Figure 5, after two days of treatment, TSG (10^−3^, 10^−4^, and 10^−5^ mg/mL) significantly increased the OPG mRNA levels and decreased the M-CSF mRNA levels. TSG (10^−3^ and 10^−4^ mg/mL) significantly decreased the RANKL mRNA levels.

### 2.4. TSG Activated the PI3K/Akt Pathway in MC3T3-E1 Cells

**Figure 6:**
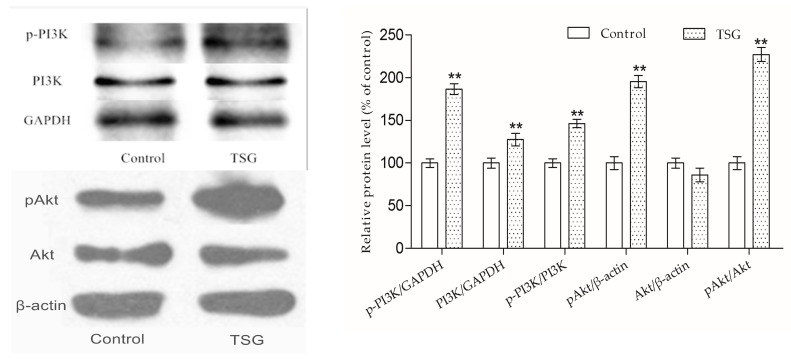
Effect of TSG on the p-PI3K, PI3k, pAkt, and Akt protein levels. Data are represented as the mean ± SD of three determinations. ** *p* < 0.01 when compared with the control group.

The Western blot strategy was used to examine TSG’s effect on the PI3K/Akt signaling pathway. As shown in Figure 6, after cells were treated with TSG (10^−4^ mg/mL) for 16 h, the p-PI3K, PI3K, and pAkt protein levels were up-regulated as were the p-PI3K/PI3K and pAkt1/Akt1 protein ratios, showing that TSG could activate the PI3K/Akt pathway in MC3T3-E1 cells. 

### 2.5. PI3K Inhibitor LY-294002 Inhibited TSG’s Effect on MC3T3-E1 Cell Proliferation

As the downstream protein of PI3K, the Akt pathway can be inhibited by the PI3K inhibitor LY-294002. In order to determine whether TSG affected osteoblast through the PI3K/AKT pathway, we used the medium containing TSG (10^−4^ mg/mL) and/or LY-294002 to treat MC3T3-E1 cells

**Figure 7:**
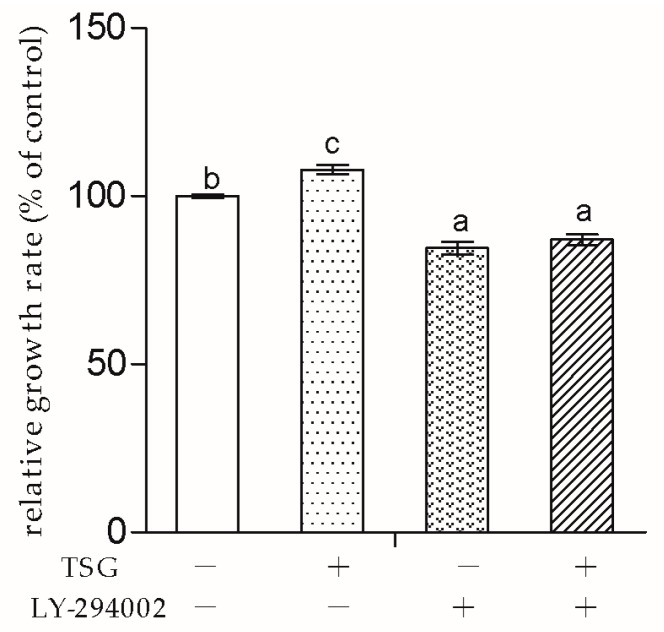
Effect of TSG and LY-294002 on the viability of the MC3T3-E1 cells. Data are represented as the mean ± SD of four determinations. Different letters indicate significant differences (*p* < 0.05).

As shown in Figure 7, After 2 days of treatment, MC3T3-E1 cell proliferation was significantly inhibited by LY-294002. Furthermore, there was no significant difference in the cell growth rates between the TSG + LY-294002 group and LY-294002 group, indicating that LY-294002 blocked TSG’s cell proliferation promotion effect.

### 2.6. LY-294002 Inhibited TSG’s Effect on MC3T3-E1 Cell Differentiation

**Figure 8:**
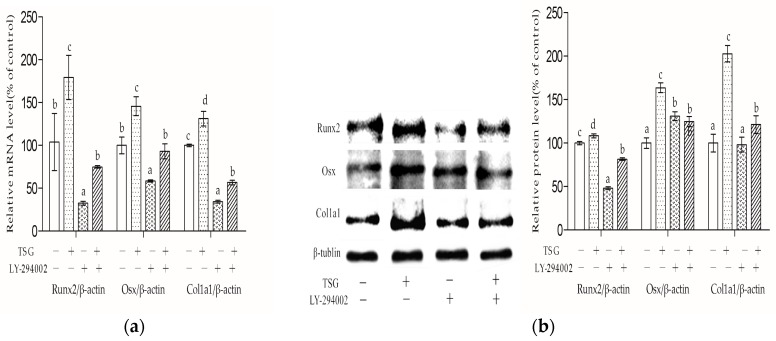
Effect of TSG and LY-294002 on Runx2, Osx, and Col1a1. (**a**) mRNA and (**b**) Protein levels of the MC3T3-E1 cells. Data are represented as the mean ± SD of three determinations. Different letters indicate significant differences (*p* < 0.05).

As shown in Figure 8a, after three days of treatment, the Runx2, Osx, and Col1a1 mRNA levels of the MC3T3-E1 cells were significantly inhibited by LY-294002. LY-294002 also inhibited the promotion effect of 10^−4^ mg/mL TSG on these mRNA levels. The mRNA levels of the TSG + LY-294002 group were significantly higher than those of the LY-294002 group. As shown in Figure 8b, TSG (10^−4^ mg/mL) up-regulated the Runx2, Osx, and Col1a1 protein levels, and LY-294002 inhibited TSG’s effect on these protein levels. LY-294002 inhibited the Runx2 protein expression, promoted the Osx protein expression, and had no significant effect on the Col1a1 protein level.

### 2.7. LY-294002 Inhibited TSG’s Effect on the OPG/RANKL Ratio and M-CSF mRNA Level

**Figure 9:**
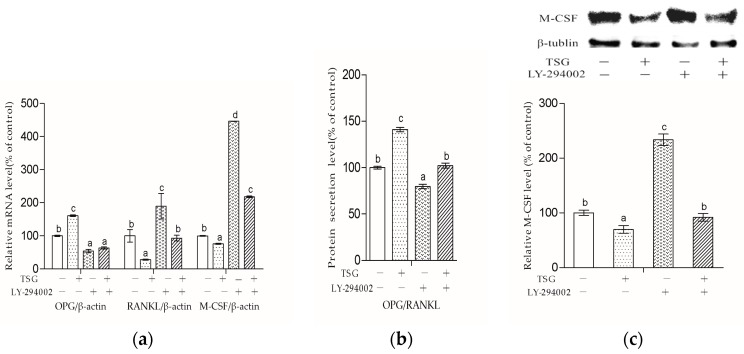
Effect of TSG and LY-294002 on (**a**) the OPG, RANKL and M-CSF mRNA levels, (**b**) the OPG/RANKL protein secretion ratios, and (**c**) the M-CSF protein levels of the MC3T3-E1 cells. Data are represented as the mean ± SD of three determinations. Different letters indicate significant differences (*p* < 0.05).

As shown in Figure 9a, after three days of treatment, LY-294002 significantly inhibited the promotion effect of 10^−4^ mg/mL TSG on the OPG mRNA level and the inhibition effect on the RANKL and M-CSF mRNA levels. There was no significant difference in the OPG levels between the LY-294002 group and TSG + LY-294002 group, but the RANKL and M-CSF mRNA levels of the TSG + LY-294002 group were obviously lower than those of the LY-294002 group. As shown in Figure 9b, after three days of treatment, TSG up-regulated the OPG/RANKL protein secretion ratio of the MC3T3-E1 cells. In addition, LY-294002 significantly inhibited TSG’s effect on the OPG/RANKL ratio, and the OPG/RANKL ratio of the TSG + LY-294002 group was significantly higher than that of the LY-294002 group. As shown in Figure 9c, after three days of treatment, TSG down-regulated the M-CSF protein level, and LY-294002 up-regulated the M-CSF level. LY-294002 inhibited TSG’s effect on M-CSF protein expression. However, the M-CSF protein level of the TSG + LY-294002 group was obviously lower than that of the LY-294002 group.

## 3. Discussion

According to traditional Chinese medicine theories, kidney essence deficiency causes brittleness of the bone, and kidney-reinforcing herbs could ameliorate osteoporosis [3]. Recent studies have demonstrated that some active ingredients in these herbs have the ability to promote bone-formation [15]. TSG—the main and unique constituent of the kidney-reinforcing herb, RPMP—also exhibited a protective effect on osteoblasts [6,7,8]. However, the mechanism of TSG’s action on osteoblasts remains unclear.

Osteoblasts create and maintain skeletal architecture in two ways: depositing bone matrix and regulating osteoclasts [9]. In this study, we measured the activity and function of pre-osteoblastic MC3T3-E1 cells treated with TSG, then tested whether TSG affected the osteoblasts through the PI3K/Akt pathway.

The MTT assay showed that 10^−3^–10^−5^ mg/mL TSG promoted MC3T3-E1 cell proliferation, and this effect was more remarkable at three days than at two days, suggesting that TSG has a protective effect on MC3T3-E1 cell viability in a time-dependent manner. Osteoblasts originate in mesenchymal stem cells. Previous studies have demonstrated that TSG could enhance the cell viability of human dental pulp stem cells [16] and rat mesenchymal stem cells [6]. Our results were consistent with the previously reported data, suggesting that TSG could protect the activity of osteoblast lineage cells. Cell proliferation is closely associated with cell cycle. According to the PI staining results, TSG up-regulated the number of MC3T3-E1 cells at the S phase, indicating that cell DNA synthesis was accelerated. Taken together, TSG could promote MC3T3-E1 cell proliferation by promoting DNA synthesis.

In the presence of differentiation transcription factors such as Runx2 and Osx, pre-osteoblasts are directed to immature osteoblasts, which express bone matrix protein genes [10]. Col1a1 is the major protein in bone matrix, which provides a structure for bone mineral to be deposited on [9]. According to the results, the Runx2, Osx, and Col1a1 mRNA transcription of MC3T3-E1 cells were up-regulated by TSG. TSG (10^−3^ mg/mL) had a more significant effect on Runx2 and Col1a1 mRNA levels than 10^−4^ mg/mL TSG, and a TSG of 10^−4^ mg/mL showed a stronger effect than 10^−5^ mg/mL TSG. There was no significant difference in the Osx mRNA levels between the 10^−3^ mg/mL and 10^−4^ mg/mL TSG groups, but they both showed a better effect than 10^−5^ mg/mL TSG. Therefore, in this study, TSG in a higher concentration exhibited a better differentiation induction effect, indicating that TSG may regulate osteoblast differentiation related genes in a concentration-dependent manner. In the osteoblast differentiation process, Osx acts as a downstream gene of Runx2 [17], and Col1a1 can be regulated by both Runx2 and Osx [10,18]. Hence, the promotion effect of TSG on the Runx2 mRNA level benefits the elevation of Osx and Col1a1 mRNA levels. Similarly, a previous study reported that TSG increased the mRNA levels of alkaline phosphatase (ALP), collagen, and osteocalcin of MC3T3-E1 cells under oxidative stress [7], and TSG promoted the osteogenic differentiation of MSCs [6]. Thus, TSG may have the ability to increase bone mass by promoting osteoblast lineage cell differentiation and bone matrix synthesis.

The bone remodeling process consists of osteoclasts removing bone and osteoblasts building new bone [2]. Osteoblasts produce cytokines such as OPG, RANKL, and M-CSF to affect osteoclasts. RANKL can bind to the receptor activator of the NF-κB (RANK) on the osteoclast and its precursors, then direct osteoclast differentiation, fusion, activation, and survival [19]. Meanwhile, OPG acts as a decoy receptor of RANKL and thus has the ability to inhibit a combination of RANKL and RANK [19]. Nowadays, some drugs including denosumab also exhibit an anti-osteoporosis effect by inhibiting the RANKL-RANK combination [2]. In this study, we demonstrated that 10^−3^ and 10^−4^ mg/mL TSG could increase the OPG mRNA level and decrease the RANKL mRNA level. As the OPG/RANKL ratio is a key determinant in the regulation of osteoclastic bone resorption [19], TSG may indirectly inhibit osteoclastogenesis and osteoclastic bone resorption by up-regulating the OPG/RANKL ratio. Aside from RANKL-RANK signaling, M-CSF–c-Fms signaling is another essential signaling pathway for osteoclasts [11]. By binding to c-Fms on osteoclast lineage cells, M-CSF could promote osteoclast viability and differentiation [11]. TSG (10^−3^–10^−5^ mg/mL) decreased the M-CSF mRNA level of MC3T3-E1 cells, which also suggests that TSG could inhibit osteoclastic bone resorption by regulating osteoblasts.

According to the results, 10^−3^ and 10^−4^ mg/mL TSG had a significant effect on MC3T3-E1 cell proliferation, differentiation, and OPG/RANKL/M-CSF transcription, and 10^−5^ mg/mL TSG had the above functions, except for influencing Osx and RANKL transcription. We chose TSG in the concentration of 10^−4^ mg/mL to conduct the following experiments.

An increasing amount of evidence has confirmed that PI3K/Akt is an essential pathway in bone formation. Akt1 and akt2 deficient mice died shortly after birth, Akt1 knockout mice exhibited impairment of bone formation, and Akt1 deficiency inhibited osteoblast survival and differentiation [14,20,21]. Previous studies have proven that TSG activates the PI3K/Akt pathway in mouse primary astrocytes and rat adrenal gland pheochromocytoma cells [22,23]. However, there is little evidence related to whether TSG could activate the PI3K/Akt pathway in osteoblast lineage cells. According to previous studies, PI3K is composed of an adapter/regulatory subunit (p85) and a catalytic subunit (p110) [24]. In this study, the p-PI3K/PI3K protein level was up-regulated under TSG treatment, showing that PI3K could be activated by TSG. The activated PI3K (pPI3K) converted phosphatidylinositole-4,5 bisphosphonate (PI(4,5)P2) into PI(3,4,5)P3, which could phosphorylate Akt to pAkt [24]. The pAkt phosphorylates serine and/or threonine of downstream substrates, conducting various cell activities [25]. Akt1 is the major Akt isoform in bone [14]. We measured the p-PI3K/PI3K and pAkt1/Akt1 protein levels of the MC3T3-E1 cells, and the results showed that TSG could activate the PI3K/Akt signaling pathway in MC3T3-E1 cells.

Next, to explore whether TSG affected MC3T3-E1 cells through the PI3K/Akt pathway, we used LY-294002 as the pathway inhibitor. In this study, LY-294002 not only down-regulated MC3T3-E1 cell viability, but also inhibited TSG’s effect on cell proliferation. The results indicated that the promotion effect of TSG on MC3T3-E1 cell proliferation was realized via the activation of PI3K/Akt.

LY-294002 inhibited the Runx2, Osx, and Col1a1 mRNA levels of the MC3T3-E1 cells, suggesting that LY-294002 inhibited osteoblast differentiation. These results were in line with previous studies, which reported that Akt1 and Akt2 double knocked-out mice exhibited impaired bone development [21], pAkt promoted the osteogenic activity of Runx2 and Osx [26,27], and LY-294002 could inhibit osteoblast differentiation by decreasing the mRNA levels of transcription factors such as Runx2 and Osx [28]. LY-294002 could also inhibit Runx2 protein expression. However, LY-294002 exhibited a promotion effect on Osx protein expression and had no significant effect on Col1a1 protein expression, which were different to the mRNA results. The reason may be because protein expression is regulated by multiple factors besides mRNA, or that the changes of protein expression appear later than mRNA level changes. In addition, TSG had a promotion effect on the Runx2, Osx, and Col1a1 mRNA and protein levels, and the effect was inhibited by LY-294002, implying that TSG promotes osteoblast differentiation through the PI3K/Akt pathway.

Figure 9 shows that LY-294002 down-regulated the OPG level and up-regulated the RANKL and M-CSF levels, suggesting that LY-294002 promotes osteoblast-inducing osteoclastic bone-resorption. In addition, LY 294002 inhibited TSG-induced OPG from increasing and RANKL from decreasing respectively, and the effect of TSG on M-CSF was also inhibited by LY-294002. Therefore, the PI3K/Akt pathway is involved in TSG’s effect on proteins, which affects osteoclast activity and differentiation.

However, in this study, LY-294002 could not totally block TSG’s effect on the Runx2, Osx, Col1a1, RANKL, and M-CSF levels in the MC3T3-E1 cells. The reason might be that the effect of TSG on MC3T3-E1 cells can be realized through other pathways. In our previous study, the herb, *Fructus Ligustri Lucidi*, and its active ingredients could affect MC3T3-E1 cells via multiple pathways [29,30]. Furthermore, there is cross-communication between the PI3K/Akt pathway and other pathways such as the MAPK pathway and JNK pathway [31]. Considering the fact that TSG could affect pathways such as Akt, AMPK, CaMKII, ERK1/2, SIRT1, NF-κB, CREB, and Nrf2 in some other kinds of cells [32], TSG’s effect on MC3T3-E1 cells may also be realized by multiple pathways. However, whether there are other pathways involved in TSG’s action on osteoblasts needs further study.

Except for its excellent curative effects and few side-effects, there are some limitations in treating patients with herbs such as the limited methods of administration. Compared with the herbs, the active natural products may possess similar regulative effects and fewer adverse reactions. They are more suitable for mass production and extensive application, and can become reliable sources for drug development [15]. As the unique compound of the kidney-reinforcing herb RPMP, TSG has the potential to be an effective agent for osteoporosis treatment. In this study, we found that TSG could promote osteoblast activity and differentiation, which benefits bone formation. On the other hand, TSG indirectly inhibited osteoclast activity and differentiation by regulating osteoblasts and thus inhibiting bone resorption. Therefore, TSG has a dual regulatory effect on osteoblasts and osteoclasts, indicating that TSG may be useful for patients who are suffering from bone diseases that are caused by deficient bone formation and/or excessive bone resorption such as osteoporosis. Furthermore, it is demonstrated that TSG affected pre-osteoblasts through the PI3K/Akt pathway. Since Akt deficiency could cause osteopenia with a low turnover state [14], TSG may be more suitable for osteoporosis patients with a deficiently activated PI3K/Akt.

## 4. Materials and Methods

### 4.1. Materials

TSG (purity = 94.7%) was obtained from the National Institutes for Food and Drug Control (Beijing, China). Fetal bovine serum (FBS) and alpha modification of Eagle’s minimum essential medium (α-MEM) medium were purchased from Hyclone (Logan, UT, USA). Methyl thiazolyl tetrazolium (MTT) and LY-294002 were purchased from Sigma Aldrich (St. Louis, MO, USA). The total RNA extraction kit was purchased from Aidlab (Beijing, China). The cell cycle analysis kit and materials for Western blot were purchased from Beyotime Biotechnology (Nantong, Jiangsu, China). Reverse transcriptase and qPCR Mix were purchased from TransGen Biotech (Beijing, China). Akt1 antibody was purchased from Signalway Antibody (College Park, MD, USA). PAkt1 (ser473) antibody was purchased from Cell Signaling Technology (Danvers, MA, USA). P-PI3K (p85) antibody was purchased from EnoGene (Nanjing, China). PI3K (p85), M-CSF, Osx, and Col1a1 antibodies were purchased from Wanleibio (Shenyang, China). The Runx2 antibody was purchased from Cusabio (Wuhan, China). GAPDH, β-actin, and β-tublin antibodies were purchased from Abclonal (Wuhan, China). OPG and RANKL ELISA kits were purchased from R&D Systems (Minneapolis, MN, USA).

### 4.2. Cell Culture

MC3T3-E1 cells were purchased from the Institute of Basic Medical Sciences Cell Resource Center of the Chinese Academy of Medical Sciences (Beijing, China). Cells were cultured in a α-MEM medium containing 10% inactivated FBS, 100 U/mL penicillin, and 100 μg/mL streptomycin in a humidified incubator with 5% CO_2_ in the atmosphere at 37 °C. For the cell viability assay and cell cycle distribution assay, the medium was then changed to a α-MEM medium containing 5% FBS, 100 U/mL penicillin, and 100 μg/mL streptomycin with different concentrations of TSG and/or LY-294002. For fluorescence quantitative real-time PCR (FQ-PCR), Western blot, and ELISA, the medium was then changed to a α-MEM medium containing 10 mM β-glycerophosphate, 50 mg/mL ascorbic acid, 5% FBS, 100 U/mL penicillin, and 100 μg/mL streptomycin with different concentrations of TSG and/or LY-294002.

### 4.3. Cell Proliferation Assay

MC3T3-E1 cells were seeded in 96-well plates at a density of 5 × 10^3^ cells/well and cultured until 80% confluence. Then, they were treated with different concentrations of TSG and/or LY-294002 (20 μM) for 2 days and 3 days. Subsequently, 20 μL MTT (5 mg/mL) was added to each well. After 4 h, the supernatant in each well was changed to 100 μL dimethyl sulfoxide (DMSO). Then, the plates were shaken for 15 min, and the absorbance value of each well was measured by a MK3 Microplate Reader (Thermo, Waltham, MA, USA) at a wavelength of 570 nm minus 630 nm. The relative cell growth rate = the absorbance value of the treated cells/the absorbance of the control cells × 100%.

### 4.4. Cell Cycle Distribution Assay

MC3T3-E1 cells were seeded in 6-well plates at a density of 2 × 10^5^ cells/well and cultured until 80% confluence. Then, they were treated with different concentrations of TSG (0, 10^−3^, 10^−4^, and 10^−5^ mg/mL) for 1 day, after being cultured in a α-MEM medium without FBS for 12 h. According to the manufacturer’s instructions, the cells were collected and fixed with 1 mL ice-cold 70% ethanol at 4 °C for 4 h, then washed twice in PBS and re-suspended in a buffer containing PI and RNase. After incubation at 4 °C for 30 min, the cell cycle distribution was measured by a FACS Calibur flow cytometer (BD Biosciences, San Jose, CA, USA). The results were analyzed by NovoExpress software (ACEA Biosciences, San Diego, CA, USA).

### 4.5. FQ-PCR Analysis

MC3T3-E1 cells were seeded in 6-well plates at a density of 2 × 10^5^ cells/well and cultured until 80% confluence. Then, they were treated with different concentrations of TSG and/or LY-294002. The total cellular RNA was extracted with an EASYspin Plus Tissue and Cell RNA Rapid Extraction Kit, then reverse transcripted by the TransScript One-Step gDNA Removal and cDNA Synthesis SuperMix according to the manufacturer’s instructions. Specific primers were synthesized by Sangon Biotech (Shanghai, China). Primer sequences were: Runx2 (Forward, 5′-CTTCACAAATCCTCCCCAAG-3′; Reverse, 5′-GAATGCGCCCTAAATCACTG-3′); Osx (Forward, 5′-CTTCCCAATCCTATTTGCCGTTT-3′; Reverse, 5′-CGGCCAGGTTACTAACACCAATCT-3′); Col1a1 (Forward, 5′-CACCCTCAAGAGCCTGAGTC-3′; Reverse, 5′-CAGACGGCTGAGTAGGGAAC-3′); OPG (Forward, 5′-CAGAGAAGCCACGCAAAAGTG-3′; Reverse, 5′-AGCTGTGTCTCCGTTTTATCCT-3′), RANKL (Forward, 5′-GATGAAAGGAGGGAGCACG-3′; Reverse, 5′-GCAGGGAAGGGTTGGACAC-3′), M-CSF (Forward, 5′-GTGTCAGAACACTGTAGCCAC-3′; Reverse, 5′-TCAAAGGCAATCTGGCATGAAG-3′); and β-actin (catalog number PMM02, Sangon Biotech, Shanghai, China). The PCR reaction in each well was performed using TransStart Green qPCR SuperMix by Applied Biosystems StepOnePlus Real-Time PCR Systems. The PCR conditions were 95 °C for 30 s, followed by 95 °C for 5 s, 60 °C for 15 s, and 72 °C for 10 s for 45 cycles. The 2^−^^△△Ct^ method was applied to calculate the relative gene expression.

### 4.6. Western Blot Analysis

MC3T3-E1 cells were seeded in 6-well plates at a density of 2 × 10^5^ cells/well and cultured until 80% confluence. Then, they were treated with TSG (0 and 10^−4^ mg/mL) for 16 h before they were lysed in Radio Immunoprecipitation Assay (RIPA) Lysis Buffer containing fresh protease inhibitor phenylmethanesulfonyl fluoride (PMSF) at 4 °C. The total protein concentrations were quantified by a Bicinchoninic Acid Protein Assay Kit (Beyotime, Beijing, China). Equal quantities of protein from each group were separated on 10% sodium dodecyl sulfate-polyacrylamice gel electrophoresis (SDS-PAGE) gels and transferred to polyvinylidene fluoride (PVDF) membranes. The membranes were blocked in TBST (Tris-Buffered Saline and Tween-20 buffer) containing 5% BSA at room temperature for 2 h, prior to incubation with primary antibodies overnight at 4 °C. Then, the membranes were washed by TBST and incubated with horseradish peroxidase-conjugated secondary antibody at room temperature for 1 h. The results were visualized by an electrochemiluminescence detection kit by Tanon 5200 (Shanghai, China).

### 4.7. Enzyme-Linked Immunosorbent Assay (ELISA)

MC3T3-E1 cells were seeded in 6-well plates at a density of 2 × 10^5^ cells/well and cultured until 80% confluence. Then, they were treated with different concentrations of TSG and/or LY-294002 for 3 days. Subsequently, the cell culture supernatant of each group was measured using OPG and RANKL ELISA Kits according to the manufacturer’s instructions.

### 4.8. Statistical Analysis

All experimental results were carried out at least in triplicate. Data were expressed as the mean ± standard deviation (SD). One-way ANOVA statistical analysis was performed, followed by a Turkey’s test for multiple comparisons, if necessary. In all cases, *p* < 0.05 was considered statistically significant.

## 5. Conclusions

In conclusion, TSG could promote MC3T3-E1 cell viability and differentiation, and influence proteins that regulate osteoclast activity and differentiation. The above effect of TSG is related to the PI3K/Akt pathway. TSG merits further investigation as a potential therapeutic agent for osteoporosis treatment.
